# Enhancement in Curie Temperature of Yttrium Iron Garnet by Doping with Neodymium

**DOI:** 10.3390/ma11091652

**Published:** 2018-09-07

**Authors:** Esperanza Baños-López, Félix Sánchez-De Jesús, Claudia A. Cortés-Escobedo, Arturo Barba-Pingarrón, Ana María Bolarín-Miró

**Affiliations:** 1 Área Académica de Ciencias de la Tierra y Materiales, Universidad Autónoma del Estado de Hidalgo, Mineral de la Reforma, Hidalgo 42184, Mexico; esperanza_banoslo@hotmail.com (E.B.-L.); fsanchez@uaeh.edu.mx (F.S.-D.J.); 2 Centro de Investigación e Innovación Tecnológica, Instituto Politécnico Nacional, Azcapotzalco, Ciudad de México 02250, Mexico; claudia.alicia.cortes@gmail.com; 3 Centro de Ingeniería de Superficies y Acabados (CENISA), Facultad de Ingeniería, UNAM, Circuito Exterior, Ciudad Universitaria, Ciudad de México 04510, Mexico; arbapin5@gmail.com

**Keywords:** Nd*_x_*Y_3−*x*_Fe_5_O_12_, rare earth doped YIG, yttrium iron garnet, Y_3_Fe_5_O_12_, Curie temperature

## Abstract

The effect of the substitution of Y^3+^ by Nd^3+^ on the structural and magnetic properties of neodymium-doped yttrium iron garnet, Nd*_x_*Y_3−*x*_Fe_5_O_12_ with *x* in the range of 0–2.5, is presented. Oxide powders of Fe_2_O_3_, Nd_2_O_3_, and Y_2_O_3_ were mixed in a stoichiometric ratio and milled for 5 h using high-energy ball milling, before being uniaxially pressed at 900 MPa and annealed at 1373 K for 2 h to obtain Nd*_x_*Y_3−*x*_Fe_5_O_12_ (0 ≤ *x* ≤ 2.5). It was found that the mechanical milling of oxides followed by annealing promotes the complete structural formation of the garnet structure. Additionally, the X-ray diffraction patterns confirm the complete introduction of Nd^3+^ into the garnet structure with a neodymium doping concentration (*x*) of 0–2.0, which causes a consistent increment in the lattice parameters with the Nd^3+^ content. When *x* is higher than 2.0, the yttrium orthoferrite is the predominant phase. Besides, the magnetic results reveal an increase in the Curie temperature (583 K) as the amount of Nd^3+^ increases, while there was enhanced saturation magnetization as well as modified remanence and coercivity with respect to non-doped YIG.

## 1. Introduction

Yttrium iron garnet, Y_3_Fe_5_O_12_ (YIG) that contain only trivalent ions, has been receiving attention for a long time due to its remarkable properties, such as high electrical resistivity (*ρ* = 10^8^ Ωm), low dielectric losses (tan δ = 1 × 10^−4^), high relative permittivity (ε_r_ = 20–100), medium saturation magnetization (*M*_s_ = 26 emu/g), and low coercivity (*H*_c_ = 17 Oe) [1,2,3,4]. 

The ferrimagnetic garnet, which has secondary symmetry, crystallizes in a cubic structure (space group *Ia3d* (O_h_^10^) with eight formula units and three sub-lattices. Of these sub-lattices, one is occupied by Y^3+^ diamagnetic ions (or rare earth ions) in dodecahedral sites {24c} according to the Wyckoff notation. The second is occupied by Fe^3+^ magnetic ions that distribute in octahedral 16a sites, which are distorted along one of the three fold axes. This trigonal axis coincides with the [111] direction. Finally, the third tetrahedral site also contains Fe^3+^ ions (24d) [5,6,7,8]. The substitution of Y^3+^ by magnetic rare earth ions promotes the formation of a new magnetic sub-lattice, which in turn causes the material to have a compensation point below room temperature [9,10]. Furthermore, due to the incorporation of doping cations into the structure of YIG, this causes changes in the magnetic, structural, and magneto-optical properties [11].

In order to understand and adjust almost any intrinsic parameter of a garnet, such as specific magnetization and coercivity, several works have substituted trivalent yttrium cations by different rare earth elements: Ce^3+^, Dy^3+^, Gd^3+^, Sm^3+^, La^3+^, including Nd^3+^ and Pr^3+^ [12,13,14]. These materials were achieved by using different synthesis methods, such as solid-state reaction, sol–gel, micro emulsion, etc. [15,16]. Among all the methods available for synthesizing YIG, the high-energy ball milling followed by thermal annealing is chosen for its simplicity and versatility [17,18]. 

Among other trivalent cations, Nd^3+^ has received special interest due to its magneto-optical properties [10] as it has the same electronic charge than yttrium +3 and thus, it does not promote the electronic hopping caused by the creation of oxygen vacancies, which are undesirable for dielectric materials. Neodymium is a light rare-earth cation, with a magnetic moment of 3.62 µB [14]. Its configuration ([Xe] 4f^3^5d^1^6s^2^) has a low 5d energy level, which subsequently results in low 4f–5d transition energy. Hence, substituting Nd^3+^ in yttrium iron garnet (YIG) could improve their magneto-optical properties [10], bandwidth efficiency [19], and dielectric properties due to the increase in spontaneous polarization. Besides, the Nd^3+^ substitution could modify the values of magnetization due to ferromagnetic coupling with the total magnetic moment of iron. This would increase the Curie temperature (*T*_c_) and hence, ensure less variation of magnetic properties with temperature (at *T* < *T*_c_), which is usually present when Y^3+^ is substituted by a trivalent light rare earth cation [20]. In addition, the introduction of Nd^3+^ could decrease the sintering temperature below 1373 K, extending their applications in low-temperature co-fired ceramics (LTCC) [21].

According to Hernández-Gómez et al. [22], the substitution of Y^3+^ by Nd^3+^ alters the magnetic properties of YIG. However, the maximum concentrations of Nd^3+^ that can be introduced into the YIG structure in order to maintain the garnet structure are *x* = 2.0 and *x* = 1.6 for the air and CO_2_ sintering atmospheres, respectively. Fratello et al. [23] successfully synthesized neodymium iron garnets by liquid phase epitaxy (LPE) at about 1173 K. They obtained films of Nd with the following characteristics YIG with a lattice parameter of 12.596 Å, Curie temperature of 567 K and a saturation magnetization of around 30 emu/g at room temperature. Komori et al. [24] synthesized the single crystals of neodymium iron garnet by low-temperature liquid phase epitaxy on Sm_3_(ScGa)_5_O_12_ and described the crystal structure garnet, in which the Nd^3+^ substituent cations were coordinated by eight oxygen atoms in distorted dodecahedrons. Arun et al. [25] studied the synthesis of Nd*_x_*Y_3−*x*_Fe_5_O_12_ by the sol–gel auto-combustion method. They described the crystallization process (garnet formation) and the Curie temperature of Nd*_x_*Y_3−*x*_Fe_5_O_12_ in the range of 527–560 K for Nd^3+^ doping concentrations of 0–1.5. 

Although there are several papers focusing on Nd*_x_*Y_3−*x*_Fe_5_O_12_ materials, there is still interest in the study of higher concentrations of Nd^3+^ cations. In this present work, we studied Nd^3+^ concentrations of 0–2.5 in the garnet structure and their effect on magnetic properties, such as: specific magnetization, coercivity and Curie temperature. In addition to the above-mentioned studies, no research about high-energy ball milling for the synthesis of this material has been conducted. We estimate that mechano-chemical processing allows us to easily introduce Nd^3+^ in dodecahedral {24c} sites in the iron garnet structure, enhancing their magnetic properties. Furthermore, we used a short milling time in order to avoid the milling medium contamination and a low annealing temperature, which made the overall process more efficient [17,18,20]. Besides, we expect changes in the Curie temperature, because it depends on the Fe^3+^_(a)_-O^2-^-Fe^3+^_(d)_ super-exchange interaction [15], which can be affected by the mechano-chemical process. As the magnetic moment of Nd^3+^ cations in the sub lattice {24c} shows ferromagnetic coupling with the total magnetic moment of iron, it could increase the Curie temperature of the Nd*_x_*Y_3−*x*_Fe_5_O_12_.

Therefore, in this present study, the powders of Nd*_x_*Y_3−*x*_Fe_5_O_12_ with different compositions (with *x* ≤ 2.5) were produced by high-energy ball milling from oxide powders, which was followed by pressing and annealing. The effect of different concentrations of Nd^3+^ on the crystal structure, Curie temperature, and magnetic properties were systematically evaluated.

## 2. Materials and Methods 

The powders of Nd*_x_*Y_3−*x*_Fe_5_O_12_ (0 ≤ *x* ≤ 2.5) were obtained from stoichiometric mixtures of Fe_2_O_3_ (Sigma Aldrich, St. Louis, MO, USA, 99% purity), Nd_2_O_3_ (Sigma Aldrich, St. Louis, MO, USA, 99.8% purity) and Y_2_O_3_ (Sigma Aldrich, St. Louis, MO, USA, 99.9% purity), according to the equation:

(1) $$x{\mathrm{Nd}}_{2}O_{3}+\left(3-x\right)Y_{2}O_{3}+5{\mathrm{Fe}}_{2}O_{3}+\to 2{\mathrm{Nd}}_{x}Y_{3-x}{\mathrm{Fe}}_{5}O_{12}$$

A total of 5 g of the starting mixture was placed together with steel balls with a diameter of 1.27 cm (ball-to-powder weight ratio of 10:1) in a cylindrical steel vial (50 cm^3^). Mixtures were milled for 5 h using a shaker mixer mill (SPEX model 8000D, SPEX^®^ SamplePrep, Metuchen, NJ, USA) at room temperature and in air atmosphere. 

These experimental conditions were selected according to previously described procedures [17,18]. In order to prevent excessive heating of the vials, the experiments were carried out by alternating 90 min of milling with 30 min of rest. Subsequently, the milled powders with different neodymium doping concentrations (0 ≤ *x* ≤ 2.5) were uniaxially pressed at 900 MPa and annealed at 1373 K for 2 h in air atmosphere at a heating rate of 10 K/min. 

X-ray diffraction technique (XRD) was used to determine the crystal structure and phases present, which was conducted using an diffractometer (Model Equinox 2000, Inel Inc., Stratham, NH, USA) in the 2θ range of 20°–80°. This was equipped with a CoKα_1_ radiation source with λ = 1.78901 Å and a germanium monochromator at 30 kV and 20 mA. In addition, the crystallographic data were obtained from the inorganic crystal structure database (ICSD). Rietveld refinements were carried out on the X-ray diffraction patterns using the MAUD (Materials Analysis Using Diffraction) software (Version 2.26, Trento, Italy) to obtain the percentages of phases, crystallite sizes and the root mean square microstrain (μs) of the materials synthesized, which is a local deviation of d-spacings from the average value caused by local defects [26]. Morphology and particle size were qualitatively evaluated by means of scanning electron microscopy (Model JSM-6300, JEOL, Tokio, Japan). For the detection of the Nd–O and Fe–O bond vibrations, chemical characterization was performed by the Fourier transform infrared (FT-IR) spectroscopy technique, using a Perkin Elmer, Lowell, MA, USA, Spectrum GX equipment, in the wavenumber range of 400–1000 cm^−1^. The samples were contained within a KBr matrix. Furthermore, magnetization studies were performed at room temperature, using a vibrating sample magnetometer (Model EV7, MicroSense, Lowell, MA, USA) and applying a maximum field of ±18 kOe. The Curie temperature was determined by means of a temperature scan test under a magnetic field of 5 kOe for all the Nd doping concentrations when the value of the derivative of the magnetization curve is at its minimum with respect to temperature. The Curie temperature was taken as the intersection point with the temperature axis of the tangent to the magnetization curve with the most negative slope.

## 3. Results and Discussion

**Figure 1:**
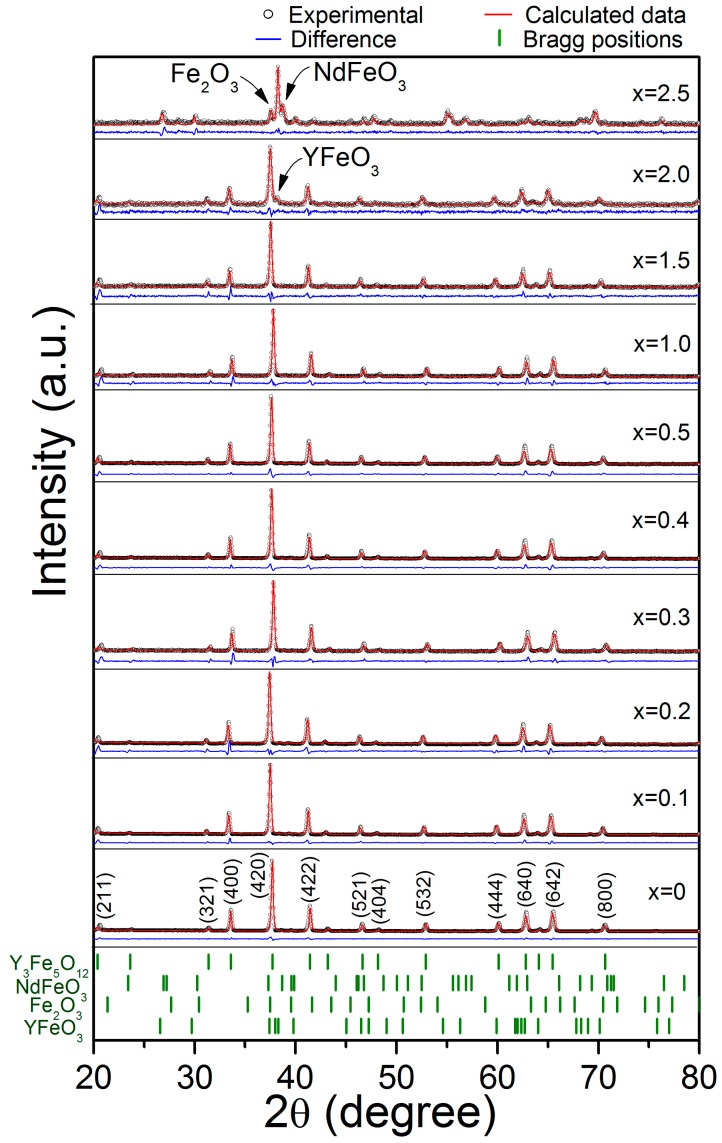
X-ray diffraction patterns and Rietveld refinement of the Nd*_x_*Y_3−*x*_Fe_5_O_12_ (0 ≤ *x* ≤ 2.5) samples mechanically milled for 5 h, pressed at 900 MPa and annealed at 1373 K for 2 h.

In Figure 1 are shown the XRD patterns for the Nd*_x_*Y_3−*x*_Fe_5_O_12_ (0 ≤ *x* ≤ 2.5) samples obtained by milling for 5 h and sintering at 1373 K for 2 h. For *x* < 2, we confirmed the complete formation of Nd*_x_*Y_3−*x*_Fe_5_O_12_ according with the ICSD #1008628 (Ia3d), which did not have peaks corresponding to the precursors (oxides). Therefore, the reaction shown in Equation (1) was completed.

In Figure 1, for a higher concentration of Nd^3+^ (*x* ≥ 2), we observed the formation of orthoferrite phases, which included: yttrium orthoferrite, YFeO_3_, ICSD #2101386 (*Pnma*); neodymium orthoferrite, NdFeO_3_, ICSD #280090 (*Pnma*); and hematite, Fe_2_O_3_, ICSD #5910082 (R-3cH). The formation of the orthoferrite phases instead of a garnet phase is due to the limit of the solubility of neodymium into the YIG structure. Despite the formation of YFeO_3_ and Fe_2_O_3_ phases, for the Nd concentration *x* = 2, it is possible to say we extended the solubility limit for neodymium doping by means of assisted mechanical milling compared to the results reported by T. Arun et al. [25] found that the maximum solubility of the Nd^3+^ in Nd*_x_*Y_3−*x*_Fe_5_O_12_ is *x* = 1.0. This increase in the solubility limit is attributed to the synthesis method, because the milling process produces an increase in the free energy of the system. The energy increase is mainly due to the storage of energy by means of lattice defects introduced by mechanical deformation and interfacial energy. Therefore, the particles become mechanically activated for chemical reactions during the annealing processes. In addition, Figure 1 shows the Rietveld refinement of the Nd*_x_*Y_3−*x*_Fe_5_O_12_ (0 ≤ *x* ≤ 2.5) samples, with the results confirming the successful synthesis of pure garnet phase for *x* < 2. 

Additionally, a distortion of the crystal structure is observed as a broadening of the diffraction peaks. This is due to the strain induced in the lattice by the neodymium substitution at yttrium positions. This causes changes in the lattice parameters due to the differences between the ionic radii of Nd^3+^ (1.109 Å) and Y^3+^ (1.019 Å) for the dodecahedral position, which has a coordination number of VIII [27].

In order to quantify the detected phases and the accumulated microstrain (μs) induced by neodymium doping, a Rietveld refinement of the XRD patterns of sintered pellets was performed; the results are presented in Table 1. As expected, whether the microstrain or lattice parameter increases with the level of neodymium due to the effect of distortion on the internal energy of the unit cell.

Table 1 presents the results of Rietveld refinement of the Nd*_x_*Y_3−*x*_Fe_5_O_12_ (0 ≤ *x* ≤ 2.5) samples from the X-ray diffraction patterns, which were discussed previously and shown in Figure 1. As observed in Table 1, the formation of a garnet structure for *x* ≤ 2 is confirmed. Furthermore, there is a slight variation of the lattice parameter (a), microstrain (μs), and decrease in crystallite size (*D*_m_) after an increase in the doping concentration (*x*). This can be attributed to the bigger size of the neodymium ion, Nd^3+^ compared to the yttrium ion, Y^3+^, which causes enough strain to reduce grain sizes and increases surface energy [23]. According to these results, we expect a simultaneous modification in the values of magnetization through the modification of the interchange interaction between the magnetic ions (iron) in octahedral and tetrahedral sites. In Table 1, χ^2^ demonstrated a good adjustment of the refinement.

**Figure 2:**
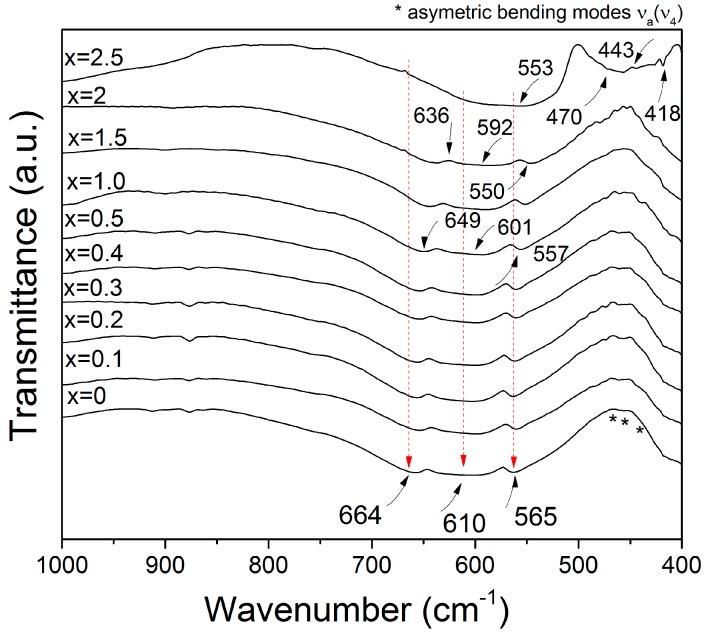
FT-IR spectra of Nd*_x_*Y_3−*x*_Fe_5_O_12_ samples recorded at room temperature.

Figure 2 shows the FT-IR transmittance spectra at room temperature in the wavenumber range of 1000–400 cm^−1^ for the Nd*_x_*Y_3−*x*_Fe_5_O_12_ samples (0 ≤ *x* ≤ 2.5) milled for 5 h and annealed at 1373 K for 2 h. As can be observed, for the non-doped sample (*x* = 0), the FT-IR spectra shows three intense vibrations corresponding to the tetrahedral sites, Fe–O bond and υ_3_ (664, 610, and 565 cm^−1^). Furthermore, a strong band at 400 cm^−1^ attributed to the isolated octahedral characteristic of the YIG was identified. The results are consistent with the space group O_h_^10^ (*Ia3d*) of YIG, which exhibits 17 triply degenerate T_1u_ modes that are active in the IR region [4,10]. Besides, there are no significant changes in bands and intensity for the absorption bands for doped samples (0.1 ≤ *x* ≤ 2.0). 

At higher concentrations (*x* > 2), a shift to the right was observed, while the asymmetric stretching modes of the tetrahedron also increases its intensity for doped samples. These facts are attributed to the distortion of the tetrahedron by the presence of Nd^3+^ at the dodecahedral site in the garnet structure. It occurs when the lattice size is increased by the introduction of neodymium ion as substitute for yttrium ion [10], and also, it may be caused by the method of preparation, which is namely mechanical activation annealing, as this causes the deformation of the structure. 

For *x* = 2.5, we observed the vibrations of the Nd–O bonds corresponding to NdFeO_3_ at 553 cm^−1^ and the band of the Fe_2_O_3_ at 443 cm^−1^. Furthermore, we observed that the vibrations at 470 cm^−1^ and 418 cm^−1^ corresponded to YFeO_3_ of the Fe–O bonds [10]. We confirmed that the garnet structure does not form in doping concentrations higher than *x* > 2. In addition, there was an asymmetrical stretching vibration of the Fe–O tetrahedral groups at 636, 592, and 550 cm^−1^, which are due to the introduction of Nd^3+^ cations into the garnet-type Nd_2_YFe_5_O_12_ [28]. Furthermore, we observed vibrations of the NdY_2_Fe_5_O_12_ sample at 649, 601, and 557 cm^−1^ for NdY_2_Fe_5_O_12._ These results are in agreement with the X-ray diffraction patterns. The FT-IR spectra from the sample doped with a high content of Nd^3+^ (*x* = 2.5) does not show the typical vibration modes of garnet structure at 664, 610, and 565 cm^−1^ as the crystal structure is orthorhombic for this composition. 

**Figure 3:**
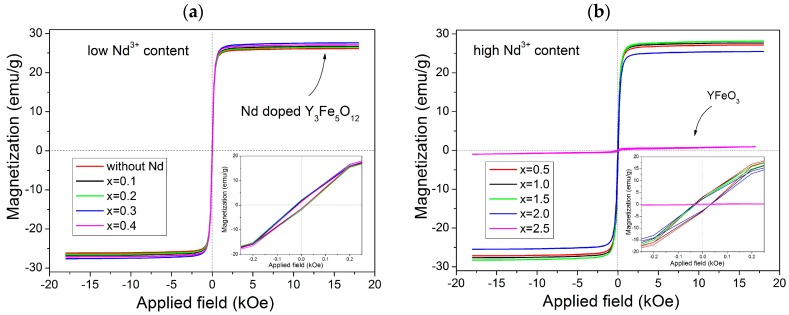
M-H loops of the Nd*_x_*Y_3−*x*_Fe_5_O_12_ for different values of *x*: (**a**) 0–0.4 and (**b**) 0.5–2.5 recorded at room temperature.

In order to describe the magnetic behavior of the synthesized materials, Figure 3 displays a field-dependent magnetization *M*(H) data measured at room temperature (magnetic hysteresis loops) with *x* = 0–0.4 in Figure 3a and *x* = 0.5–2.5 in Figure 3b. Two different behaviors are observed. The first shows a ferrimagnetic order where the specific magnetic saturation (*M*_s_), which is around 26 emu/g, is reached by applying a magnetic field close to 2 kOe for *x* ≤ 2 (Figure 3a). The second shows a weak ferrimagnetic material for *x* = 2.5 with a specific magnetization of 1 emu/g (Figure 3b). These behaviors are attributed to the different crystal structures observed in XRD patterns in Figure 1 as there was a garnet structure for *x* ≤ 2 and orthoferrite structure for *x* = 2.5. Besides, it can be observed that the value of specific magnetization for *x* ≤ 2 is high, which was attributed to the interactions of super exchange of the (24d) sites. These sites are not influenced by the Nd^3+^ and instead depend on the angles between iron ions. However, the reduced magnetizations in 16a sites can be attributed to the lower strength of the Fe–O bond in tetrahedral sites. The substitution of Y^3+^ by Nd^3+^ improved the magnetic order of the structure by ferromagnetic coupling with the total magnetic moment of iron. However, when *x* = 2.5, there is a marked change in behavior.

**Figure 4:**
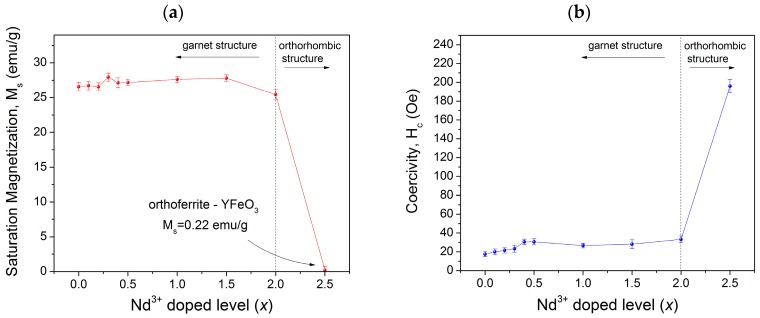
(**a**) Specific magnetization (*M*_s_); and (**b**) coercivity of the Nd*_x_*Y_3−*x*_Fe_5_O_12_ samples (0 ≤ *x* ≤ 2.5) milled for 5 h and annealed at 1373 K.

In order to analyze the magnetic hysteresis loops shown in Figure 3, the variation of specific saturation magnetization (*M*_s_) and coercivity (*H*_c_) versus neodymium content (*x*) is presented in Figure 4. As observed, we obtained 17.5 Oe of coercivity for the non-doped sample, which is slightly lower than samples synthesized by other methods [11,12,13,14]. This result can be due to the lower crystallite size achieved by high-energy ball milling and the formation of a complete solid solution of yttrium iron garnet. In addition, we detected a systematic increase in the coercivity with an increase in neodymium concentration. This is attributed mainly to the microstrain in crystal structure of YIG since crystal deformation and grain boundaries are related to the movement of the domain wall. Thus, additional energy is required in the form of a larger applied field to overcome the decrease in the energy of the wall and to release it from grain boundaries. This results in an increase in the values of *H*_c_ for the multi-domain structure, where the regions of uniform magnetization are separated by domain walls [3]. 

The magnetic properties of the garnets depend on several parameters, such as the composition of the material, the particle size, method and temperature of synthesis [8,9,20]. In the studied case, large size particles were obtained (~3 µm) with low coercivity (˂40 Oe). From these measurements, it is possible to establish that the substitution of Y^3+^ by Nd^3+^ increased the magnetic anisotropy in the lattice with a positive small contribution of Nd ions to spontaneous magnetization.

For the specific saturation magnetization, the values of *M*_s_ are around 26 emu/g when *x* is 0–0.15 (Figure 4). Meanwhile, for *x* = 2.0, a slight decrease in the specific magnetization is produced, which reaches 25.4 emu/g due to the formation of YFeO_3_ and Fe_2_O_3_ phases. Finally, for *x* = 2.5, the specific magnetization falls down to 1 emu/g. This is when we obtained a mixture of yttrium orthoferrite (89 wt.%), neodymium orthoferrite (1 wt.%), and iron (III) oxide (10 wt.%), which showed the magnetic behavior corresponding to the mixture of oxides without formation of a solid solution (0 ≤ *x* ≤ 2.0) [29,30,31]. 

The garnet structure has three sub-lattices—namely, octahedral [16a], tetrahedral (24d), and dodecahedral {24c}—that are aligned along the [111] direction. According to the Neél theory, for doped heavy rare earth ions (moment and spin are related by a negative g factor) being added into garnet, the {24c} sub lattice is formed by the Y^3+^ ions, which are coupled anti-ferromagnetically to the total iron moment [8,15]. The total magnetic moment (*M*_YIG_) for the garnet structure of YIG is defined by the equation

(2) $$M_{\mathrm{YIG}}=\left(M_{d}-M_{a}\right)-M_{c}$$

where *M_d_*, *M_a_*, and *M_c_* are the magnetic moments of cations in tetrahedral (*d*), octahedral [*a*], and dodecahedral sites {*c*}, respectively. According to theoretical results from Wolf, rare earth ions, such as Ce^3+^, Pr^3+^, and Nd^3+^, have positive parallel alignment at the effective moments formed by Fe^3+^ ions [16]. The Nd^3+^ is a magnetic ion (3.62 μ_B_) that is a substitute for Y^3+^, which is a diamagnetic ion (by steric aspects, it cannot substitute Fe^3+^). Hence, we expect an increase in the saturation magnetization of yttrium–neodymium iron garnet at room temperature by increasing Nd^3+^ concentration. However, the experimental results shows that the specific magnetization remains nearly constant with a change in the neodymium content. This behavior is attributed to the synthesis method and other factors that promote changes in the magnetic order of the cationic moments due to structural distortions modifying the net magnetic moment. 

**Figure 5:**
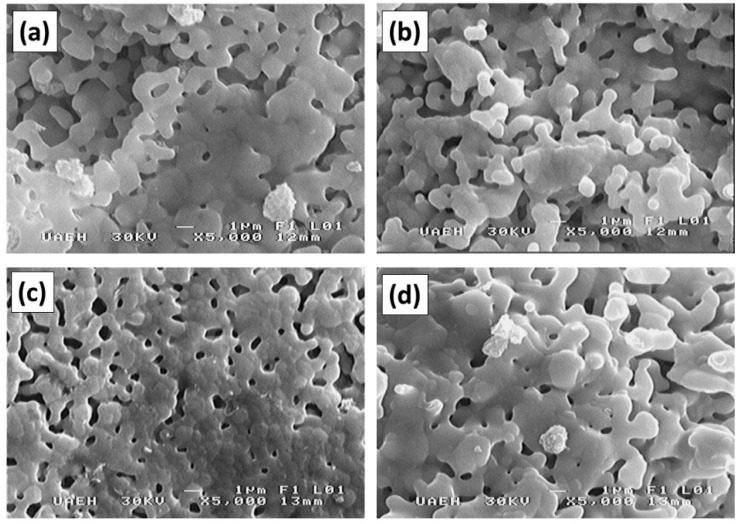
SEM micrographs of milled powder annealed at 1373 K for 2 h for obtaining Nd*_x_*Y_3−*x*_Fe_5_O_12_ with different values of *x*: (**a**) *x* = 0, (**b**) *x* = 0.5, (**c**) *x* = 1.0, and (**d**) *x* = 2.0.

Figure 5 shows the particle morphologies from the SEM of the powder mixtures milled for 5 h for obtaining Nd*_x_*Y_3−*x*_Fe_5_O_12_ (0 ≤ *x* ≤ 2.0) and annealed at 1373 K for 2 h. In this figure, we observed particles of approximately 3 µm in diameter with a nearly uniform size distribution. Additionally, we saw the formation of agglomerates covered by smaller particles with a smooth surface, which had an irregular and rounded morphology (almost identical for all samples). This indicates that the concentration of Nd^3+^ into the YIG does not modify the morphology of the particles, although changes could occur in crystallite sizes.

**Figure 6:**
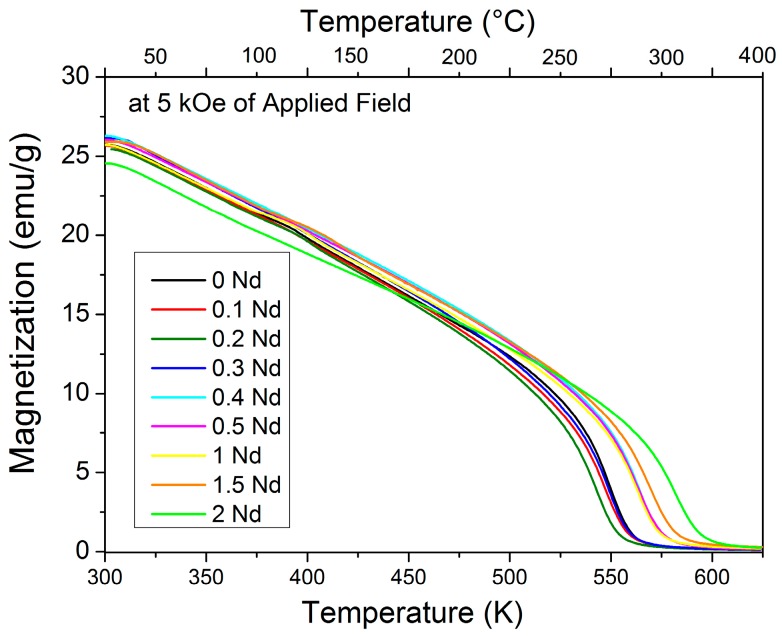
Temperature dependence of magnetization of the Nd*_x_*Y_3−*x*_Fe_5_O_12_ (0 ≤ *x* ≤ 2.0) at 5 kOe of applied field.

Finally, we evaluated the effect of adding Nd^3+^ ion to the YIG crystal structure and its relationship with the Curie temperature, with the results shown in Figure 6. For temperatures higher than *T*_c_, a complete random orientation of the magnetic moments takes place (paramagnetic state), which results in zero magnetization. For this reason, the increase in *T*_c_ can be explained by the increasing concentration of Nd^3+^ ions due to the positive contribution of Nd ions that increased the magnetic moment of samples. 

The *T*_c_ of the un-doped sample obtained was 549 K, which is consistent with other authors [4,15]. By increasing the Nd^3+^ content, an increase in *T*_c_ was found as *T*_c_ increased from 549 K for the non-doped YIG to 583 K for doped sample with *x* = 2.0. Hence, this can be explained by the major contribution of the 5d orbitals of Nd^3+^, which is larger than the 6s. As previously mentioned, Nd^3+^ increased the magnetic moment and enhanced the *T*_c_ (up to 583 K) for high contents of neodymium (*x* = 2). 

**Figure 7:**
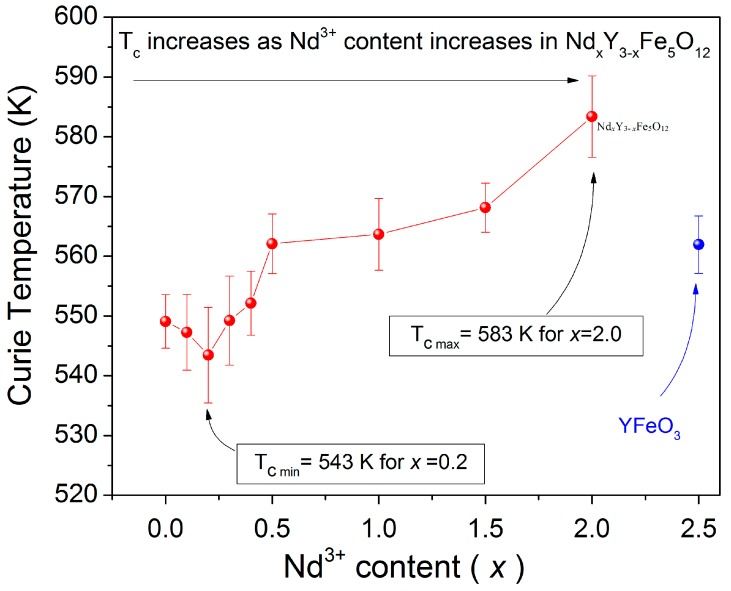
Curie temperature (*T*_c_) of the Nd*_x_*Y_3−*x*_Fe_5_O_12_ (0 ≤ *x* < 2.5).

Therefore, the values of *T*_c_ in the samples are attributed to the interactions between magnetic moments, which also are related to the lattice parameter and grain size of the samples. Thus, the values of magnetization decrease when the temperature increases because the order of magnetic moments of atoms is randomized. In fact, the importance of the substitution is to modulate the magnetic and magneto-optic properties of YIG. In the studied material, the *T*_c_ decreased from 547 to 543 K for *x* = 0.1 and 0.2, respectively, which is shown in Figure 7. This is due to changes in the length and angle of Fe^3+^_(a)_-O^2-^-Fe^3+^_(d)_ so the interaction and effective magnetic moments formed by Fe^3+^ decrease. This also changes the entropy of the material with a low Nd^3+^ content. After this point, there is a consistent increase in *T*_c_ as the doping level increases, which reaches a maximum of 583 K at *x* = 2.0 where the formation of orthoferrite and hematite phases is observed. 

The results reveal that Nd*_x_*Y_3−*x*_Fe_5_O_12_ (0 ≤ *x* ≤ 2.0) shows simultaneously high values of magnetization and high Curie temperature. Finally, for *x* = 2.5, where mainly the orthoferrite structure is detected by XRD, the Curie temperature falls down to 562 K. This result is not presented in Figure 6 since the specific magnetization at 5 kOe is 1 emu/g and would be observed as a horizontal line on the same axis scale. 

Another factor explaining the behavior of the Curie temperature is the contribution of the 5d orbitals of Nd^3+^. As Y^3+^ is a diamagnetic ion, the exchange interaction is zero, meaning that the joined structure causes distortion of the crystal lattice. Taking into account all the above-mentioned points, it is possible to modulate some properties—such as *M*_s_, *H*_c_, and *T*_c_—for applications that demand specific values for these parameters.

## 4. Conclusions

Neodymium-doped yttrium iron garnet, Nd*_x_*Y_3−*x*_Fe_5_O_12_ (0 ≤ *x* ≤ 2), has been successfully prepared by high energy ball milling for 5 h, followed by annealing at 1373 K for 2 h. This temperature is around 200 K lower than the temperature required to obtain a garnet structure by means of solid state reaction. X-ray diffraction and Rietveld refinement confirmed that a high neodymium-doped garnet material is synthesized, while the garnet structure is retained for almost all the doping levels (*x* ≤ 2) of the prepared powders. We observed the formation of orthoferrite phases of Nd and Y with Nd^3+^ contents of *x* > 2 and small amounts of hematite. FT-IR analysis confirms the structural changes detected with an increase in Nd^3+^, which is used as a substituent cation. All the studied samples show ferrimagnetic behavior attributed to the intrinsic structure, garnet or orthorhombic phases. The saturation magnetization obtained for the doped samples is in the range of 25.4–27.9 emu/g, while the coercivity was between 17 and 33 Oe. Both values are in good agreement with the magnetic parameters obtained by similar methods for pure yttrium iron garnet and represent important magnetic contributions. 

Moreover, integrating the neodymium ions into the structure of YIG increases the Curie temperature up to 583 K for *x* = 2, with high magnetic parameters, such as specific magnetization and coercivity. The presence of Nd^3+^ as the doping cation primarily affects the Curie temperature of the neodymium-doped yttrium iron garnet, while the specific magnetization remains constant. This extends the range of potential applications of these ferrites due to their tunable magnetic properties.

## Figures and Tables

**Table 1:** Rietveld refinement parameters for Nd_x_Y_3−x_Fe_5_O_12_ (0 ≤ *x* ≤ 2.5) after milling and annealing.

Nd Doped Level*, x*	Phase	Space Group	Phase wt.%	Dm (Å)	Lattice Parameters (Å)	μs	χ^2^
0	Y_3_Fe_5_O_12_	*Ia3d*	100	1900(100)	a = 12.3718(3)	0.001300(5)	1.13
0.1	Nd_0.1_Y_2.9_Fe_5_O_12_	*Ia3d*	100	1900(100)	a = 12.3764(9)	0.001200(4)	1.07
0.2	Nd_0.2_Y_2.8_Fe_5_O_12_	*Ia3d*	100	1600(90)	a = 12.3966(1)	0.001300(5)	1.11
0.3	Nd_0.3_Y_2.7_Fe_5_O_12_	*Ia3d*	100	1700(20)	a = 12.3752(3)	0.001400(4)	1.05
0.4	Nd_0.4_Y_2.6_Fe_5_O_12_	*Ia3d*	100	1750(50)	a = 12.3909(3)	0.001300(5)	0.99
0.5	Nd_0.5_Y_2.5_Fe_5_O_12_	*Ia3d*	100	1650(90)	a = 12.3944(3)	0.001300(6)	1.00
1.0	Nd_1.0_Y_2.0_Fe_5_O_12_	*Ia3d*	100	1570(50)	a = 12.3953(3)	0.001100(8)	0.80
1.5	Nd_1.5_Y_1.5_Fe_5_O_12_	*Ia3d*	100	1360(40)	a = 12.4492(3)	0.001200(6)	0.84
2.0	Nd_2.0_Y_3_Fe_5_O_12_	*Ia3d*	81.7(8)	730(17)	a = 12.4557(5)	0.001300(7)	0.89
	YFeO_3_	*Pnma*	14.0(6)	915(0)	a = 5.588(3) b = 7.595(4) c = 5.274(2)	0.00097(0)	
	Fe_2_O_3_	*Pnma*	4(1)	859(0)	a = 5.000(3), c = 13.600(9)	0.00090(0)	
2.5	YFeO_3_	*Pnma*	89.0(1)	915(26)	a = 5.558(5), b = 7.569(6), c = 5.239(4)	0.00097(0)	0.84
	NdFeO_3_	*Pnma*	1.0(5)	1000(0)	a = 5.589(6), b = 7.7619(7), c = 5.4489(6)	0.00060(6)	
	Fe_2_O_3_	*R-3cH*	10.0(9)	860(180)	a = 5.017(2), c = 13.673(9)	0.0009(5)	

*D*_m_: crystallite size; μs: microstrain.
